# Supplementary material for: Are carbon emissions trading and green financial instruments synergistic? -Comprehensive quantitative research based on content analysis
Source: PLoS One. 2024 Mar 7;19(3):e0298601. doi: 10.1371/journal.pone.0298601 (PMC10919601; doi:10.1371/journal.pone.0298601)
Supplement: S1 Data — (ZIP) [file pone.0298601.s001.zip › Data/地级市Z_F工作报告.zip!/╒π╜¡/─■▓¿╩╨/└┤╫╘╣1⁄2╓┌║┼í╛┬φ┐╦╩2╛▌═°í┐_2015.docx]

各位代表：

　　现在，我代表市人民政府向大会报告工作，请予审议，并请市政协委员和其他列席人员提出意见。

　　一、2014年工作回顾

　　2014年是实施“十二五”规划的关键一年，也是全面深化改革的开局之年。市政府认真贯彻党的十八大和十八届三中、四中全会精神，全面落实省委“八八战略”和市委“六个加快”战略，大力实施“双驱动四治理”决策部署和“经济社会转型发展三年行动计划”，着力稳增长、促改革、调结构、惠民生、防风险，各项工作取得了扎实成效。

　　一年来，经济扩总量、促增量、提质量取得新进展。实现地区生产总值7602.5亿元，增长7.6%。完成一般公共预算收入860.6亿元，增长8.6%。实现固定资产投资3989.5亿元，增长16.6%。完成规上工业增加值2540.2亿元，增长7.4%，其中规上装备制造业增长11.1%。城市现代化、国际化、品质化实现新提升。轨道交通1号线一期建成运营，步入立体交通新时代。港口集装箱吞吐量跃升到全球第五位，增幅位居大陆主要港口首位。成功承办亚太经合组织首次高官会议和中国—中东欧国家经贸促进部长级会议，启动建设全国首个保险创新综合示范区，中意宁波生态园批准设立，宁波杭州湾新区升格为国家级开发区，“三江六岸”滨江休闲带建设加快推进。政府透明度位居省会城市、计划单列市首位。城乡居民收入水平、居住环境、生活条件得到新改善。城乡居民收入增长快于经济增长，农村居民收入增长快于城镇居民收入增长。“五水共治”、“四边三化”、“三改一拆”、交通拥堵治理深入推进。城乡居民基础养老金待遇标准提高11.8%，城乡居民低保标准提高12%，职工最低工资标准平均提高12%。政府十方面民生实事全面完成。

　　一年来，我们主要做了五个方面的工作：

　　（一）抓项目、强支撑，“三年行动计划”全面实施。把推进“三年行动计划”作为政府工作的重中之重，狠抓协调服务和要素保障，强化动态监测和工作督查，实施重大项目419个，完成投资1362.4亿元，超额完成年度任务。在“五水共治”方面，全面推行“河长制”，治理垃圾河174条、黑臭河613公里，甬江流域防洪工程全线开工，五大应急泵站在汛期前建成投用，农村生活污水治理和钦寸水库水源工程进展顺利，“治水强基”项目投资额和完成率位居全省第一。在现代产业培育方面，海越新材料、吉利春晓整车等项目建成投产，上海大众宁波基地扩建、吉利—沃尔沃中国设计及试验中心等项目开工建设，华侨城、华强·中华复兴文化园一期、宁南贸易物流区等项目加快推进。在城市功能提升方面，电子商务“一城两区一中心”、浙商回归总部基地、轨道交通2号线一期、铁路宁波站综合交通枢纽等项目稳步推进，轨道交通3号线一期、东苑立交快速化改造等项目开工建设，南北环快速路基本建成。在民生福祉改善方面，市妇儿医院北部院区建成投用，宁波杭州湾医院、杭州湾协同创新园区一期、宁波工程学院新校区、市花卉园林示范区等项目加快实施。在发展基础强化方面，中国电信云计算宁波分中心等项目稳步实施，铁路货运北站建成投用，三门湾大桥及接线、机场三期综合交通枢纽等项目开工建设。

　　（二）抓改革、建机制，各项改革和政府自身建设有效推进。把改革作为政府工作的主线，深化重点领域和关键环节改革。加快简政放权。实施“四张清单一张网”改革，市级部门行政权力事项精简幅度达到59%，基本建设项目联审联办机制实现常态化运作，市政府采购中心进驻公共资源交易平台。制定政府职能转变和机构改革方案，优化行政机构职能配置。推进重点领域改革。探索要素差别化配置改革，实施工业企业单位资源占用产出绩效评价，城镇低效用地再开发工作得到加强。激发社会投资创业活力，鼓励民间资本进入社会事业和特许经营领域，推进商事登记制度改革，新设内资企业数和注册资本分别增长28%和82%。加强重大战略谋划。开展行政区划优化布局研究，拟定参与国家“一带一路”建设“1+4”方案，谋划港口经济圈和国际海洋生态科技城建设，“十三五”规划前期工作有序启动。深化依法行政。严格落实人大决议决定，自觉接受人大法律监督和工作监督，主动接受政协民主监督、人民群众监督和新闻舆论监督。建立重点工作与人大对接、与政协协商制度，完善政府部门与人大、政协工作机构对口联系制度，探索建立政府部门与民主党派、工商联对口协商制度。承办代表建议582件、政协提案558件。加强建章立制，严格行政执法，提交人大审议地方性法规议案5件，制定修改政府规章8件。切实改进作风。推动作风建设制度化、规范化、常态化，完善领导干部基层调研、联系服务企业和重大项目分级协调机制，加强政府工作目标管理和绩效考评。严格建设资金和政府债务管理，强化审计监督和行政监察，完善廉政风险防控机制。“三公”经费支出下降30.3%。

　　（三）抓产业、调结构，经济转型发展步伐加快。坚持综合施策、多管齐下，打好转型发展“组合拳”。强化创新驱动发展。推动创新机构、创新企业、创新人才集聚和创新成果转化，新材料科技城、高新区“一区多园”、“千人计划”余姚产业园等平台加快建设。出台高校毕业生购房补贴政策，人才安居环境得到进一步改善。引进共建科研机构56家，新增市级以上企业工程（技术）中心和研究院177家，引进“3315”高端创新创业团队27个。发明专利授权量增长26.1%，全社会研究与试验发展经费支出占地区生产总值比重预计达到2.35%。加快工业转型升级。推进“四换三名三创”工程，开展工业自动化（智能化）成套装备改造试点，规上工业企业全员劳动生产率提高9.9%。加强“两化”融合，培育信息经济，实施市民卡提质扩面和手机支付试点，入选“中国十大智慧城市”。发展战略性新兴产业，推进南车宁波产业基地、北仑高端装备产业园、杭州湾智能装备产业园建设，新能源汽车等产业发展迅速。注重质量引领、内涵发展，“质优宁波”建设全面启动。建筑业转型发展步伐加快，产值增幅位居全省首位。培育壮大城市经济。发展信息、文化、教育、健康等新兴消费，扶持“月光经济”、电子商务、休闲旅游等产业发展，网络零售额、旅游总收入分别增长82.1%和12%，社会消费品零售总额增长13.5%。加快国际贸易、港航物流等产业发展，举办中东欧国家特色产品展、国内外“宁波周”等活动，原油贸易实现“零突破”，宁波大宗商品交易所交易额实现翻番增长。启动金融生态示范区建设，金融机构本外币存贷款余额分别增长5.5%和9.4%，直接融资总额增长87%。提升发展现代农业。推进农业“两区”建设，健全农业现代经营体系和农产品质量安全检测体系，实施渔场修复振兴计划和“一打三整治”行动，加快林特产业发展。新建现代农业园区40个、标准化粮食生产功能区10.1万亩。生态公益林补偿标准提高到每亩35元。

　　（四）抓统筹、重品质，港口城市建设提速增效。加强城乡规划、建设和管理，深入实施城市国际化行动纲要。完善城乡功能。扎实推进城市总规修改、土地利用规划调整和宁波都市区规划编制工作，开展“多规融合”试点。加快重大功能区块建设，宁波杭州湾新区、梅山产业集聚区开发建设势头强劲，东部新城、南部新城、镇海新城、东钱湖旅游度假区等区域形象进一步提升，都市副中心、卫星城、中心镇、特色镇等规划建设取得新进展。深化农村产权制度改革、农村股份合作社改革和土地经营权流转机制改革，幸福美丽新家园建设和相对欠发达地区发展步伐加快。优化城乡环境。加强环境专项治理和执法监管，淘汰落后产能企业505家、黄标车6.5万辆，“禁燃区”面积达到1053平方公里，“三改”和拆违分别完成年度任务的324%和164%。推进“两心一轴、三江六岸”核心景观带建设，启动中山路综合整治工程，加强城市精细化管理，获评“中国十佳户外广告管理示范城市”，中心城区新增公共绿地170公顷。深化对外开放。加强重点开发区域整合提升，推进综合保税区申报和象保合作区建设，南部滨海新区挂牌成立，新加坡宁波丰树现代创新基地签约落户。深化跨境贸易电子商务和进口贸易便利化试点，启动关检协作“三个一”通关模式，推动上海自贸区政策在我市复制实施，加快“世贸通”等外贸综合服务平台建设。梅山、穿山港区创建成为国际卫生港，梅山口岸扩大开放通过国家验收。实现外贸自营进出口总额1047亿美元，其中出口731.1亿美元、增长11.3%；国际服务外包执行额增长50.8%。实际利用外资40.3亿美元，浙商甬商回归项目实际到位资金增长30%，实际引进内资745.4亿元。对口支援帮扶和“山海协作”取得新成效。

　　（五）抓民生、促和谐，居民生活品质持续改善。城镇居民人均可支配收入44155元，农村居民人均可支配收入24283元，分别增长9.2%和11%。公共财政民生支出比重达到66.3%。加强就业促进工作。深化国家创业型城市和充分就业县（市）区创建，高校毕业生初次就业率达到95%，城镇新增就业17.5万人，城镇登记失业率下降到1.95%。完善社会保障体系。推进被征地人员养老保障、低标准养老保险与基本养老保险制度转换衔接，实施城乡居民大病保险办法，整合城乡医保管理体制，开展阳光医保监管平台试点，启动巨灾保险。本地居民养老保障参保率达到91.5%。推进“医养结合”养老工作，市老年疗养院建成投用。改善居民居住条件。推动房地产市场平稳健康发展，适时调整居民购房政策，新开工保障性安居工程456万平方米、39960套，解决中低收入家庭住房困难9515户。开展以成片危旧住宅区为重点的城市棚户区改造试点，启动试点项目13个、改造8491户。繁荣发展社会事业。弘扬社会主义核心价值观，推进文明城市创建，健全公共文化服务体系，举办第六届中国国际声乐比赛、首届全国文化馆年会和市民文化艺术节，中国港口博物馆、中国摄影家协会宁波艺术中心建成开放，3件作品荣获“五个一”工程奖，大运河（宁波段）申遗成功。推动学前教育公益普惠，促进普高多样优质发展，扩大“职普融通”改革试点，实施高校协同创新计划，所有县（市）区通过全国义务教育发展基本均衡评估认定。中英时尚学院获批建设，新增国（境）外合作学校120所。加快新十大医疗卫生基础设施建设，深化市县两级公立医院改革，推行家庭医生制服务，重大传染病得到有效防控。奥体中心开工建设，全民健身深入开展，我市运动员在第15届省运会上取得历史最好成绩。创新社会治理机制。深化重大决策社会稳定风险评估，建立矛盾纠纷挂牌督办机制，推进市长电话“一号通”政务热线整合，健全公共法律服务和应急管理体系，开展流动人口居住证制度改革和积分制公共服务试点，村（社区）“五多”清理深入开展，食品药品“两网五体系”建设加快推进。完善安全生产工作机制，强化重点行业领域专项整治，安全生产事故起数和死亡人数实现“十连降”。档案史志、哲学社会科学、新闻出版、计划生育、妇女儿童、残疾人和公益慈善事业稳步发展，国防动员、双拥、民族、宗教、红十字工作进一步加强，外事、港澳、对台、侨务工作取得新进展。

　　各位代表！在外部经济形势纷繁复杂、内部发展新问题新挑战持续显现的情况下，过去一年我市取得这样的成绩实属不易。这是党中央国务院、省委省政府和市委科学决策、正确领导的结果，是市人大、市政协有效监督、大力支持的结果，也是全市上下克难攻坚、共同奋斗的结果。在此，我代表市人民政府，向全市人民，向市人大代表、市政协委员，向离退休老干部，向各民主党派、工商联、人民团体和社会各界人士，向驻甬人民解放军和武警部队官兵，向“宁波帮”和帮宁波人士，以及所有关心支持宁波发展的海内外朋友们，表示衷心的感谢和崇高的敬意！

　　同时，我们也清醒地看到，我市发展中还存在不少困难和挑战，政府工作还有许多不足。经济内生增长动力不够强，战略性新兴产业、现代服务业发展不够快，地区生产总值没有完成预期目标；重点领域和关键环节改革攻坚步伐有待加快，开放合作优势需要进一步增强；新型城市化建设任重道远，基本公共服务与群众期望还有一定差距，生态环境保护力度有待加大，社会治理能力有待提高。金融、房地产和政府性债务的风险隐忧仍然存在。同时，政府职能转变有待加快。有的部门执行力、担当力不强，主动作为、协作配合不够；一些政府工作人员法治意识、改革意识、服务意识不强，办事效率不高；一些领域的消极腐败现象仍然存在。对此，我们将标本兼治、对症下药，切实加以改进。

　　二、2015年主要任务

　　2015年是全面深化改革的关键之年、全面推进法治宁波建设的开局之年、全面完成“十二五”规划的收官之年和“十三五”规划的谋划编制之年。我们将深刻认识新常态、主动适应新常态、积极引领新常态，聚焦点、抓重点、破难点，努力开创经济社会发展新局面。今年政府工作的总体要求是：全面贯彻落实党的十八大和十八届三中、四中全会精神，认真学习贯彻习近平总书记系列重要讲话精神，坚持以“八八战略”为总纲，按照“干好一三五、实现四翻番”的要求，深入实施“六个加快”战略，扎实推进“双驱动四治理”决策部署和“经济社会转型发展三年行动计划”，以建设港口经济圈为重点，积极融入和服务国家“一带一路”、长江经济带战略，着力深化改革开放，着力提质增效升级，着力城乡区域协调，着力生态环境保护，着力惠民利民便民，努力保持经济平稳健康发展和社会和谐稳定，为实现“两个基本”、建设“四好示范区”奠定更加坚实的基础。

　　建议今年经济社会发展的主要预期目标为：地区生产总值增长7.5%左右，一般公共预算收入同口径增长8%，港口集装箱吞吐量增长6%；城乡居民人均可支配收入分别增长9%和10%，城镇新增就业14.5万人，城镇登记失业率控制在3.5%以内，居民消费价格指数与国家价格调控目标衔接；节能减排指标完成省定目标任务。

　　围绕上述目标要求，重点做好六个方面的工作：

　　（一）建设港口经济圈，营造发展新优势

　　积极融入和服务国家“一带一路”、长江经济带战略。以建设港口经济圈为抓手，引领宁波—舟山港一体化、宁波都市区规划建设和现代化国际港口城市建设。提升集聚辐射带动能力，推进江海联运服务中心建设，打造全国多式联运枢纽，开展沪甬跨海大通道工程规划研究。优化“无水港”布局，建设宁波—华东地区集装箱海铁联运示范通道，构建“甬新欧”贸易物流线，促进港口腹地向长江流域、西北地区延伸拓展。深化与“一带一路”沿线城市的开放合作，申报设立国际港口合作服务组织常设机构，建立“海上丝绸之路”综合指数平台，积极筹办中国—中东欧国家投资贸易博览会和“中国航海日”活动。完善港口基础设施和集疏运网络，推进国际空港物流园区、梅山港区集装箱码头等项目建设。加快形成港口经济的核心圈、覆盖圈、辐射圈和互联互通圈，充分发挥“一带一路”桥头堡和长江经济带龙头的龙眼作用。

　　加快建设海洋经济强市。高水平、高标准规划建设国际海洋生态科技城，打造区域创新发展新高地。推进国际强港建设，健全港口物流服务体系，推动甬商所加快发展，完善航交所、甬易第三方支付等港航服务平台，大力扶持航运金融、航运保险、航运经纪、仓储物流等新兴港口服务业发展，促成东海航运保险公司开业运营。择优发展临港产业，优化功能、结构和布局，促进临港产业向产业链、价值链高端攀升。加快海洋特色产业基地、涉海产业功能区块建设，深化海域海岛产权交易改革，组建宁波海洋研究院，不断健全海洋科技支撑体系。

　　（二）推进“三年行动计划”，奠定更实发展基础

　　加强项目谋划生成。突出重大基础设施、城市经济、重大产业、工业技改、生态环保等领域，落地生成一批带动力强、质量效益好的重大项目，谋划储备一批“十三五”重大项目。做实项目前期，完善项目储备库制度，积极争取在规划布局、审批服务、要素保障等方面得到国家和省里更大支持。强化招商选资，紧盯世界500强、央企和龙头民企，加强定向招商与战略合作，大力推动浙商甬商回归创业。实施“三年行动计划”重大项目521个，完成投资1600亿元以上。固定资产投资增长12%以上。

　　加强项目高效实施。围绕提高水环境质量，开工建设桃源水厂、姚江北排等项目，加快建设农村生活污水处理工程，建成宁波北区污水处理厂二期、鄞东南沿山干河一期等项目。围绕增强现代产业引领能力，开工建设电动汽车总部基地、中船宁波装备产业园等项目，加快上海大众宁波基地扩建、南车宁波产业基地、国际金融服务中心二期等项目建设，基本建成吉利—沃尔沃中国设计及试验中心、宁波模具产业园等项目。围绕提升城市集聚辐射功能，推进新城有序开发、老城有机更新和交通网络优化，实施新一轮主干道整治提升，开工建设院士桥，建成新江桥、机场快速干道永达路连接线等项目。围绕促进社会民生改善，开工建设市图书馆新馆、煤电机组清洁排放提标改造等项目，推进城市棚户区改造，加快奥体中心、滨江大道及沿江景观等项目建设。围绕增强基础设施承载保障能力，推进电网输变电、移动光纤到户等项目建设。

　　加强项目服务保障。统筹管理规划空间、土地资源、环境容量，完善能耗总量控制政策，实施建设用地增减挂钩、城镇低效用地再开发和海涂保护开发利用，推进地下空间开发，提高投资开发强度。健全项目筹资管理制度，深化投融资体制改革，探索政府与社会资本合作模式、国有资产混合所有制等试点，扩大直接融资，推动融资主体和渠道多元化。推进项目全程服务，强化区域协同联动，完善领导干部联系服务、“红黄绿灯”管理、联合督查和考核评价制度，营造优质高效的项目投资环境。

　　（三）深化改革开放，增强发展动力

　　加大改革力度。深化行政审批制度改革，继续下放一批审批权限，优化审批流程，扩大网上审批，加强事中事后监管。推进贸易便利化改革，深化关检协作“三个一”通关和进口贸易便利化试点，加快建设进出口商品采购贸易改革示范区、梅山口岸贸易便利化示范区。稳步推进要素市场化改革，实施要素分层定价、企业分类指导和生态环境财政奖惩制度，落实生产性服务业用水、用电、用气与工业同价政策。开展国家新型城镇化综合试点，实施农业转移人口市民化、城镇化投融资和不动产统一登记制度改革。深化商事登记制度改革，推动民营企业创新发展，强化国有企业“瘦身强体”，实施涉企收费服务目录清单制，更大程度激发市场主体活力。完善督促落实机制，加强改革成效评估，切实让改革举措更好更快地转化为发展动力。

　　提高开放水平。复制推广上海自贸区实践经验，跟踪推进金融、航运、商贸等领域扩大开放。完善扩大出口综合政策，深入实施“外贸实力效益工程”，加强境外直销体系建设，扶持“世贸通”、“小树外贸通”等外贸综合服务平台发展，拓展转口贸易、离岸贸易、租赁贸易等业务，努力培育出口竞争新优势。推进进口升级行动，推进跨境贸易电子商务试点，加快汽车整车进口口岸建设，完善进口商品内销体系。外贸自营进出口总额增长5%，其中出口增长7.5%以上。统筹招商资源，发挥华侨华人、海外“宁波帮”、专业咨询服务公司等作用，构建互动共促的招商体系。实际利用外资42亿美元。推动“千军万马走出去”，培育境外生产制造、贸易营销和资源开发基地，完善境外突发事件应急机制。整合提升重点开发区域，加快中意宁波生态园、象保合作区建设，推动产业（功能）区高端化、特色化、集约化发展。加强与国际友城的交流交往，深化教育、文化、医疗、人才等领域的交流合作。拓展区域合作领域，进一步做好对口支援帮扶和“山海协作”工作。

　　（四）强化创新转型，提升产业发展

　　着力创新驱动。以平台建设为重点，推进新材料科技城“一城多园”发展，加快宁波新材料（国际）创新中心建设，引进共建诺丁汉大学宁波新材料研究院、中电科（宁波）海洋电子研究院、中船海洋装备动力研究院等重大科研机构，争创国家自主创新示范区。以协同创新为支撑，深化科技体制改革，推进科技与经济的结合，做强天使投资、科技信贷风险池，支持创业基金发展，完善公共技术服务平台，深入实施“科技领航”、“智团创业”计划，培育壮大创新型企业、创新团队和技术研究院，大力营造“大众创业、万众创新”的浓厚氛围。深入实施重大科技产业化专项，强化发明专利实际应用，加快新能源汽车应用示范试点。全社会研究与试验发展经费支出占地区生产总值比重达到2.5%，发明专利授权量增长10%。以优化创新环境为保障，加强知识产权保护，深入实施“3315计划”和海外工程师计划，新增各类人才15万人。整合财政专项资金，设立并发挥好工业与信息发展基金、海洋经济发展基金、电子商务发展基金和旅游发展基金的作用，促进产业创新转型。

　　打造工业强市。推进“质优宁波”建设，深化“四换三名三创”工程，促进工业提质增效升级。围绕新材料、新能源、新装备、节能环保、海洋高技术等重点领域，培育壮大战略性新兴产业。全力打造一批省级“特色小镇”，推动产业集聚、产业创新和产业升级。增强产业链竞争新优势，谋划实施一批补链、强链型优质项目，开工建设康龙化成生命科技产业园、中国移动宁波信息通讯产业园，加快推进“千人计划”余姚产业园建设，力争中海油大榭石化三期馏分油、中金石化技改项目竣工投产。引导中小微企业提升发展，推动现代企业制度建设，促进“个转企、小升规、规改股、股上市”，加大困难企业帮扶解困力度。综合实施工业企业单位资源占用产出绩效评价，加快块状行业整治提升，启动“十百千万”专项行动。规上工业增加值增长8%。推进新型建筑工业化、住宅产业化，加快建筑业转型发展。

　　提升发展服务经济。深化服务业综合改革试点，推动生产性、生活性服务业协同并进。培育发展总部经济、科技研发、文化创意、服务外包、中介服务、汽车服务后市场等业态，力争国家广告产业试点园区开园运营。发展壮大“月光经济”，建设一批特色夜市街区，促进商文、商旅、商娱、商体融合发展。加快建设电子商务“一城两区一中心”，健全配套服务体系，培育电商经济创新园区。大力提升旅游业发展水平，加快天一阁·月湖5A景区和东钱湖国家级旅游度假区创建，推进雪窦山佛教名山和鄞江风情小镇打造，加强慈城古县城保护开发。全面深化金融生态示范区和全国保险创新综合示范区建设，扩大城乡小额贷款保证保险，加快发展普惠金融。切实优化服务业发展环境，完善生命健康、养老服务、信息消费等产业发展政策，推动省级服务业集聚区、市级现代服务业基地建设。社会消费品零售总额增长12%。

　　推进农业现代化。创建国家级现代农业示范区，深化农业结构调整。加快农业“两区”建设，新建标准化粮食生产功能区10万亩、现代农业园区30个。健全粮食生产、流通、储备和调控体系，确保种植面积达到209万亩。推进科技兴农，发展循环农业、现代种业、乡村旅游业、农产品仓储和冷链物流业，保障农产品质量安全。培育新型农业经营主体，培养生产型、技能型、经营型农村实用人才，健全社会化为农服务体系，完善强农惠农政策，促进农民收入持续普遍较快增长。

　　建设智慧城市。发展信息经济，促进产业信息化和信息产业化互动，争创信息经济发展先行区。以智能制造为突破口，推进“两化”深度融合，实施“机联网”、“厂联网”示范工程，推动制造模式向数字化、网络化、智能化、服务化转变。建设重点行业信息技术应用公共服务平台，扶持工业信息工程公司、智慧工厂工程公司发展。完善通信基础设施，推动免费无线上网从主城区向县（市）区延伸，确保“村村通”宽带接入率达到100%。启动智慧城市运营中心、城区“E邮站”建设，发展智慧教育、智慧城管，探索智能交通管理服务系统，拓展市民卡应用范围，开展智能化气象服务，努力让城市管理更高效、居民生活更方便。

　　（五）推进新型城市化，展现城乡新面貌

　　优化都市发展格局。推进城市总规修改和土地利用规划调整，深化“多规融合”。加快宁波都市区规划建设，完善主体功能区规划，有序推进行政区划优化调整，增强市域统筹能力和区域发展活力。完善中心城区集聚辐射功能，实施三江口核心区、东部新城、中山路轴线等核心区域十大提升工程。注重地上与地下联动、区块与区块联通、老城与新城协调发展，推进道路清爽行动和交通拥堵治理，提升现代都市形象。优化“一核两翼、两带三湾”多节点网络化空间布局，提升姚北新城、慈溪文化商务区发展水平，加快宁南贸易物流区、南部滨海新区、象山大目湾新区建设步伐，形成各区域“争先促转型、合力谋发展”的生动局面。

　　完善交通网络体系。推进城市轨道交通、城际铁路建设，开工建设2号线二期、4号线和宁波至奉化城际铁路，加快3号线一期建设，确保2号线一期通车试运营、1号线二期具备通车条件。加强市域内外交通连接，推进甬金铁路、杭甬高速复线宁波段项目前期，开工建设穿山港口铁路支线和北仑支线电气化改造工程，加快机场三期扩建、三门湾大桥及接线、杭州湾跨海大桥杭甬高速连接线等项目建设，促进余慈地区交通设施互联互通。完善城市快速路网和跨江交通体系，深化“公交都市”建设和打通“断头路”专项行动，开工建设南北环快速路东延、东外环快速路改造项目，推进东苑立交快速化改造、机场路北延工程，建成投用铁路宁波站综合交通枢纽，努力使地面交通更便捷、地下交通更快捷。

　　加快城乡统筹发展。坚持城乡一体化发展，促进城市基础设施、公共服务和社会事业向农村延伸。完善镇村布局规划，深化美丽乡村建设，推进历史文化名城、名镇和名村保护发展。注重“物的新农村”和“人的新农村”建设齐头并进，推进“三村一线”创建和“百村示范千村整治”工程，创建市级全面小康村30个，启动培育中心村15个、特色村20个。加快土地经营权流转和产权交易改革，基本完成农村经济合作社股份制改革，有序推进农房“两改”，提升农村金融服务，激发农村发展活力。支持卫星城和中心镇改革发展，加大相对欠发达地区扶持力度。完善农村物流服务体系，提高城乡客运一体化水平。推动四明山区域生态发展，促进产业绿色转型和基础设施内联外接，加快污染治理、生态修复和旅游景观升级，打造“多彩山水、美丽四明、幸福老区”。

　　促进环境有效改善。全面推进“五水共治”，加快防洪排涝体系建设，深化保供水和抓节水工作，深入推动“两覆盖”、“两转型”，力争“清三河”任务提前一年完成。全面推进“四边三化”，加快“三江六岸”滨江休闲带建设，推动平原绿化、生态公益林和城区公共绿地建设。全面推进“三改一拆”，深入开展无违建创建，实现改拆各1000万平方米。全面推进“三治理一提高”行动，强化工业废气治理、粉尘扬尘污染防治、重污染行业整治和土壤污染治理，重拳打击环境违法行为，确保县（市）区中心区域“禁燃区”全覆盖，基本淘汰黄标车，中心城区垃圾分类收集覆盖面达到50%。全面推进低碳城市试点，科学划定生态红线，完善生态安全网，努力满足人民群众对清新空气、清澈水质、清洁环境的美好期待。

　　提高城市文明水平。深化文明城市创建，积极践行社会主义核心价值观。加强普法教育，培育法治文明，积极营造办事依法、遇事找法、解决问题用法、化解矛盾靠法的法治环境。彰显开放包容的城市特质，加强城市品牌形象推介，促进新老市民和谐共处，提高公共设施、公共服务的国际化和便利化水平，提升城市友好度和美誉度。加强公民道德建设和市民素质教育，完善道德荣誉体系，促进社会诚信制度化、志愿服务制度化，全面关爱社会困难群体，大力倡导“爱心宁波·尚德甬城”好风尚，让城市文明之光照亮社会每个角落。

　　（六）顺应群众期盼，优化公共服务

　　提供更优质的教育资源。发展普惠性幼儿园，提高义务教育均衡发展水平，推进普高多样化发展，实施高水平大学建设和一流学科、专业群建设工程，完善全民终身教育体系。实施技术技能人才培养提速计划，加快国家职业教育与产业协同创新试验区建设，争创全国现代职教开放示范区。加快现代学校制度建设，完善教师全员聘用、义务段教师校长交流机制，深化中考中招和新课程改革，拓展与世界知名大学合作办学，促进民办教育规范发展。加大教育基础设施投入，启动建设国家级学生综合实践基地、学生职业体验拓展中心，基本建成宁波工程学院新校区。推进百万学生餐饮安全工程，强化学校安全管理，为广大学生创造优良的学习环境。

　　提供更丰富的文化产品。深入实施文化惠民工程，提高“文化走亲”活动成效，办好中国湖泊休闲节、市民文化艺术节、“海上丝绸之路”文化周、阿拉音乐节等重大文化活动。推动文化与科技、金融、旅游、商贸深度融合，争创文化金融合作试验区，组建文化产业投资基金，鼓励民间资本参与国有文化单位改革。加快实施农村文化礼堂、公益电影放映基地、好书交换亭等一批文化项目，扎实推进江北老外滩天主教堂修缮工程，力争华强·中华复兴文化园一期开园运营。培育壮大体育产业，提升发展群众体育、竞技体育，办好首届宁波国际马拉松比赛和第17届市运会。

　　提供更高水平的医疗服务。深化医药卫生体制改革，探索药品集中采购、医院人事薪酬制度改革，完善财政拨付与医院绩效挂钩机制，建立以法人治理结构为核心的现代医院管理制度。加强医疗卫生服务资源整合，建立医疗机构联合体，构建分级诊疗体系。全面推行契约式家庭医生制服务，鼓励社会力量办医、医师多点执业。加快完善医疗卫生基础设施，扩建市中医院，新建宁波西部医院（暂名），建成投用李惠利医院东部院区。推进“云医院”建设，实施城乡居民健康卡工程，努力让居民看病更方便。

　　提供更可靠的社会保障。完善居民就业创业政策，建立面向全体劳动者的职业培训体系。推进机关事业单位养老保险制度改革，深化外来务工人员社保与职工社保制度并轨，进一步提高养老保障待遇。整合新农合和城镇居民医保制度，建立统一的城镇职工和城乡居民大病保险办法，推行大医院和基层医疗机构差别化的医保支付制度，实施基本医疗保险按病种付费管理方法，全面推进阳光医保监管平台建设。深入实施工资集体协商制度，提高劳动纠纷预防调处水平。完善住房保障体系，推进公共租赁住房和廉租住房并轨运行，深化以成片危旧住宅区为重点的城市棚户区改造，健全城镇危旧房常态化、网格化安全监管体系。建立商品房土地供应与存量消化挂钩机制，创新保障性住房房源提供模式，保持房地产市场平稳健康发展。健全新型养老服务体系，引导民办养老机构发展，完善养老服务配套产业链，更好地促进“老有所养、老有所乐”。

　　营造更和谐的社会环境。完善依法维权和矛盾化解体系，健全利益表达、协商沟通、行政裁决和救济救助机制，联动推进人民调解、行政调解和司法调解，依法处置信访问题，努力实现风险预警和矛盾化解常态化。加强公共法律服务，扩大公职律师、公司律师试点。推进多层次、多领域依法治理，强化流动人口管理服务，引导各类社会主体自我约束、自我管理。加快应急平台建设，整合各类应急资源，提高突发事件和重大自然灾害应对能力。增强社区服务功能，推广“365社区服务工作法”。构建立体化社会治安防控体系，严厉打击暴力恐怖、“两抢一盗”、非法集资、黄赌毒、逃废债等违法犯罪行为。推进“依法治安”，强化安全生产基层基础，开展安全生产隐患第三方评估，实施化工园区封闭式管理，确保安全生产形势持续稳定好转。加强国家安全和民族宗教工作。支持驻甬人民解放军和武警部队现代化建设，推进军民融合深度发展，争创全国双拥模范城“七连冠”。

　　各位代表!群众所盼就是施政所向。今年，我们将继续办好一批关注度高、涉及面广、带动力强的民生实事，让全市人民更好地共享改革发展成果。

　　三、加快建设法治政府

　　围绕经济发展新常态、改革开放新要求和人民群众新期待，市政府将出台法治政府建设实施意见，加快建设职能科学、权责法定、执法严明、公开公正、廉洁高效、守法诚信的法治政府，确保政府活动在法治框架内开展、在法治轨道上运行。

　　推进科学施政。突出民生导向，密切联系群众，谋民利、解民忧，深化民生重点领域改革，健全为民服务长效机制，推进基本公共服务均等化。健全依法决策机制，严格规范重大行政决策的公众参与、专家论证、风险评估、合法性审查、集体讨论等程序，加强决策评估和跟踪反馈，健全终身责任追究及责任倒查机制。认真评估“十二五”规划实施效果，科学谋划编制“十三五”规划，做好重大发展战略、规划和政策的制定实施工作。推进政府职能转变和机构改革，加强政府事权规范化、法制化，完善宏观调控方式，创新资源配置模式，最大限度减少政府对微观事务的管理。

　　推进依法行政。认真执行市人大及其常委会的决议、决定，加强代表建议、政协提案办理工作。自觉接受人大监督、政协监督、司法监督、社会监督和舆论监督，完善政府内部层级监督和专门监督，让人民监督权力，让权力在阳光下运行。深化“四张清单一张网”建设，推进决策公开、执行公开、管理公开、服务公开和结果公开，完善项目推进和工作协调机制，不断提高政府行政服务效能。完善行政执法体制，推动跨部门、跨领域基层综合执法，探索部门联合执法，严格规范行政执法行为，坚决纠正行政不作为、乱作为，进一步提高政府执行力、公信力。

　　推进勤廉优政。践行“三严三实”，强化党风廉政建设主体责任，推进“四风”整治和腐败惩治。加大对权力集中部门和重点岗位的监督力度，推进重点领域审计监督全覆盖，防止权力乱用滥用。深化财政体制改革，推进部门预算公开，完善建设资金和政府债务统一管理制度。加强公务员培训和监督管理，深化公务员分类制度改革，探索县以下机关公务员职务与职级并行制度，强化绩效考核和纠错问责机制。完善重点产业（功能）区及相关县（市）区目标管理考核办法，切实发挥考核的导向激励作用。厉行勤俭节约、反对铺张浪费，严格控制一般性支出，确保“三公”经费继续下降。

　　各位代表！今年的工作任务繁重而艰巨，我们肩负的使命光荣而神圣。让我们更加紧密地团结在以习近平同志为总书记的党中央周围，在省委、省政府和市委的坚强领导下，紧紧依靠全市人民，坚定信心、鼓足干劲，开拓创新、奋发有为，为顺利完成“十二五”规划确定的各项任务，为实现“两个基本”、建设“四好示范区”而努力奋斗！
